# Stay Safe and Strong: Characteristics, Roles and Emotions of Student-Produced Comics Related to Cyberbullying

**DOI:** 10.3390/ijerph19148776

**Published:** 2022-07-19

**Authors:** Consuelo Mameli, Laura Menabò, Antonella Brighi, Damiano Menin, Catherine Culbert, Jayne Hamilton, Herbert Scheithauer, Peter K. Smith, Trijntje Völlink, Roy A. Willems, Noel Purdy, Annalisa Guarini

**Affiliations:** 1 Department of Education Studies “Giovanni Maria Bertin”, University of Bologna, 40126 Bologna, Italy; 2 Department of Psychology “Renzo Canestrari”, University of Bologna, 40127 Bologna, Italy; laura.menabo@unibo.it; 3 Faculty of Education, Free University of Bolzano, 39042 Bolzano, Italy; antonella.brighi@unibz.it; 4 Department of Humanities, University of Ferrara, 44121 Ferrara, Italy; damiano.menin@unife.it; 5 Department of Psychology, Goldsmiths, University of London, London SE14 6NW, UK; cathculbert@gmail.com (C.C.); p.smith@gold.ac.uk (P.K.S.); 6 School of Social Sciences, Education and Social Work, Queen’s University, Belfast BT7 1PS, UK; jayne.hamilton@qub.ac.uk; 7 Department of Education and Psychology, Freie Universität Berlin, 14195 Berlin, Germany; hscheit@zedat.fu-berlin.de; 8 Department of Psychology, Open University of the Netherlands, 6419 AT Heerlen, The Netherlands; trijntje.vollink@ou.nl (T.V.); roy.willems@ou.nl (R.A.W.); 9 Centre for Research in Educational Underachievement, Stranmillis University College, Belfast BT9 5DY, UK; n.purdy@stran.ac.uk

**Keywords:** cyberbullying, adolescents, co-participatory approach, arts-based method, comics

## Abstract

The present study aimed at giving voice to students from disadvantaged socio-economic backgrounds using a co-participatory approach. Participants were 59 adolescents (52.5% males) aged between 14 and 16 from five European countries who created ten comics to illustrate cyberbullying for a broader audience of peers. We analyzed texts and images according to four primary themes: cyberbullying episodes (types, platforms, co-occurrence with bullying), coping strategies, characters (roles, gender, and group membership), and emotions. The content analysis showed that online denigration on social media platforms was widely represented and that cyberbullying co-existed with bullying. Social strategies were frequently combined with passive and confrontational coping, up to suicide. All roles (cyberbully, cybervictim, bystander, reinforcer, defender) were portrayed among the 154 characters identified, even if victims and defenders appeared in the vignettes more often. Males, females, peers, and adults were represented in all roles. Among the 87 emotions detected, sadness was the most frequently expressed, followed by joy, surprise, anger, and fear. Emotions, mainly represented by drawings or drawings with text, were most often represented in association with cybervictims. The results are discussed in terms of their methodological and practical implications, as they emphasize the importance of valorizing young peoples’ voices in research and interventions against cyberbullying.

## 1. Introduction

Cyberbullying is a widespread issue among adolescents [1]. The prevalence rates reveal wide ranges for perpetration (6% to 46%) and victimization (14% to 57.5%), as described by studies at European [2,3] and worldwide levels [1]. Victims show decreases in self-esteem, wellbeing, and life satisfaction and increases in symptoms of depression, suicidality, anxiety, hostility/aggression, and loneliness [4,5]. Cyberbullying constitutes a public health problem with growing rates of nasty online experiences and depression in recent years [6].

Given its spread and severity, cyberbullying has been extensively investigated in the last decade [7]. However, research on this issue has mainly adopted an adult-centered approach, which basically conceives of young people as research objects [8,9,10]: children and adolescents are treated as informants, as in studies aimed at collecting information on definitions, rates, predictors, or consequences [11,12], or as beneficiaries, as in studies implementing interventions designed to reduce or prevent cyberbullying [13,14]. In both these cases, young people participate in the research process by playing a passive role. In this respect, a few scholars [15,16,17,18] have raised concerns about the exclusive use of adult-centered methods: how can researchers fully understand a phenomenon they have not directly experienced or set priorities for interventions? Young people’s understanding of cyberbullying and their experience of new technologies, preferences, values, and culture, are inevitably qualitatively different from those of adult researchers [19,20]. In light of these considerations, and consistent with Hart’s Ladder of Participation model [21], a few studies [8,10] have emphasized the need for a methodological shift towards co-participatory approaches allowing a greater valorization of children’s and adolescents’ voices and roles in research.

Another important methodological limitation is that to date, cyberbullying has been mainly studied with samples of white middle-class students [17,22], thus neglecting the perspective, and possibly the active contributions, of young people from areas of socio-economic disadvantage. Although several studies have involved young people from minority groups by investigating different forms of discriminatory cyberbullying (e.g., related to disability [23] or to minority ethnic groups, [24]), they mainly employed adult-centered quantitative methods, with few opportunities for these adolescents to have their voice heard and fully recognized [17].

To address the above-mentioned methodological gaps, in this study we used a co-participatory approach to ascertain young people’s understanding of cyberbullying through the analysis of comics produced by adolescents from areas of socio-economic disadvantage.

### 1.1. Co-Participatory Design Approaches

By and large, co-participatory approaches [9,17] require that young people are actively involved in the research process from the earliest stages, and share with researchers the power to decide on the study contents, phases, methods, and tools, assuming an agentic role. Young people also share with scholars the responsibility to produce, as well as disseminate, materials, outputs, and innovations. The use of a co-participatory approach to investigate cyberbullying has several advantages. First, it allows the depiction, from an insider perspective, of a clearer and more nuanced comprehension of the phenomenon [10,15]. Second, it increases potential usability and impact, as young people’s knowledge is crucial to grasp what elements can make policy and practice more effective [20].

Notwithstanding the lack of research using this approach, and especially the lack of studies involving young people from areas of socioeconomic disadvantage, the results of a few co-participatory studies are promising in terms of increased knowledge of cyberbullying, as well as of developing dissemination materials and designing interventions. In a study conducted by Ashktorab and Vitak [15], for instance, high school students worked with researchers to design solutions to address cyberbullying. In another study [16], high school students collaborated with researchers to develop and implement a series of whole-school activities to decrease cyberbullying-related incidents.

### 1.2. Arts-Based Methods

In co-participatory social and educational research, arts-based methods are emerging as a successful strategy to empower young people and give them greater participation opportunities in the research process [25,26,27,28]. Generally, arts-based methods cover a wide range of activities—from making photos and collages to creating sculptures or comics—allowing participants to explore their ideas, opinions, and experiences, and translate them into artistic products [29,30]. Compared to traditional qualitative methodologies such as interviews or focus groups, creative activities emphasize some of the aspects that have been mostly overlooked in previous scientific research, that is, visual information [31]. In comics-based research [30], for instance, creators produce multimodal information allowing words and pictures to interact and reinforce one another fully interdependently. By creating artistic outputs, young people can share their knowledge innovatively [29,32,33] and face emotions difficult to express in words [27,34].

Another reason for the growing success of arts-based methods is the possibility of promoting the active engagement of young participants. The playful nature of this approach [35], combined with the opportunity to use multiple communication channels [28], stimulates young people’s curiosity and involvement. This approach is crucial in studies involving children and adolescents with language difficulties, such as immigrant students or students from disadvantaged backgrounds [29,36,37,38], who commonly struggle to express their thoughts and feelings verbally.

In the scientific fields focused on bullying and cyberbullying, arts-based methods have been mostly ignored. While cartoons developed by researchers (e.g., vignettes representing peer aggression) have been used to evoke children’s understandings and definitions of these phenomena [39,40,41], very few investigations have requested young participants to actively contribute to producing artistic representations of bullying [42,43]. Other studies used comic strips or vignettes as a stimulus to tell complex stories focused on bullying and asked children to articulate those stories or complete them by creating additional drawings or cartoons [27,44,45]. To the best of our knowledge, however, no study so far has investigated young people’s representations of cyberbullying by asking them to tell their own stories through the creation of drawings, vignettes, or comics.

### 1.3. The Present Study

Prior research on cyberbullying has mainly been conducted using adult-centered methodological approaches [9], thus failing to achieve the goal of empowering young people, especially those from areas of socio-economic disadvantage [17], with a more authentically active and co-participatory role in the research process and the designing of consciousness-raising and intervention materials [10]. Furthermore, the few co-participatory studies focused on cyberbullying have primarily emphasized the verbal aspects of young participants’ ideas and perceptions [27,31]. This resulted in a limited possibility to access, by means of visual elements and representations [30], the thoughts and ideas of children and adolescents with language difficulties [29]. In the light of these premises, the overall aim of the present study was to explore how early adolescents from disadvantaged socio-economic backgrounds in Italy, England, Germany, the Netherlands, and Northern Ireland, choose to represent the phenomenon of cyberbullying through comics to be disseminated to their peers.

There were four specific aims of the study. First, we explored the nature of the different cyberbullying incidents that adolescents depicted in their comics, i.e., type of online incident, web platform, and link (if any) to offline bullying. We expected that students would represent different types of cyberbullying, describing several forms of online aggressions (verbal offensive responses and insults, visual violence victimization, impersonation and account forgery, and sexual harassment), as suggested by previous studies [1,46]. Furthermore, as previous studies indicate that cyberbullying occurs across many media platforms [47], we also expected to find various web platforms represented. Finally, we hypothesized that cyberbullying and traditional bullying episodes would be present in the same comics, confirming the overlap between the two phenomena [48,49,50].

The second aim was to explore which coping strategies adolescents represent to deal with cyberbullying. Based on previous research [51,52], we expected that students would depict different strategies: effective actions to solve the situation (e.g., asking for help from friends, teachers, and parents), passive strategies (e.g., wishing for a miracle to stop cyberbullying), and, in extreme cases, self-harming behaviors such as suicide.

Our third aim was to investigate which characters (i.e., presence) are portrayed in comics—in terms of roles, gender, and peer or adult group membership—and how often they are represented in the single vignettes making up the comics (i.e., relevance). We expected that all of the participant roles (bully, victim, reinforcer, defender, and bystander) [53] would be represented, in line with studies revealing students’ awareness of cyberbullying as a group process and a social phenomenon [12]. However, we also expected that victims’ and bullies’ roles would be overrepresented, both in terms of presence and relevance, as they are considered the main actors in cyberbullying [51]. Furthermore, we hypothesized that students would depict both males and females in different roles. In this respect the literature showed contrasting results, revealing in some cases that males were more likely to become cyberbullies, and in other cases a higher prevalence of females in perpetration (for reviews, see [1,54,55]). Concerning peer or adult group membership, the scarce literature on this topic does not allow us to make any hypotheses based on previous evidence. However, it is reasonable to assume that adults would be depicted mainly in the role of victims’ defenders, while peers would be portrayed in all the roles traditionally associated with cyberbullying.

Finally, the fourth aim was to explore which emotions young people decide to represent in the comics, their association with the portrayed participant roles, and their representation in textual messages and/or visual elements. We expected a prevalence of displeasing emotions (e.g., sadness) to highlight the negative impact of cyberbullying. Previous studies on bullying [42,43] and cyberbullying [56,57] showed that sadness and other negative emotions are usually identified in victims, while expressions of moral disengagement are commonly found in bullies. Thus, we expected that cybervictims would be characterized by negative emotions and cyberbullies by positive ones. Concerning bystanders, reinforcers, and defenders, our study was explorative. Furthermore, due to the nature of our material, we expected that emotions would be represented both in terms of visual elements (e.g., facial expressions) and textual messages (e.g., captions). However, given that in comics visual data generally dominate over textual data, and given the language difficulties of our participants, we expected visual information to be more prevalent. On this point, it should be noted that linguistic difficulties were not tested directly in our participants, although they were reported by researchers present during group-work sessions aimed at creating comics. Moreover, in a preliminary survey conducted in the same schools chosen for the co-participatory work on comics, more than 19% of students reported having learning difficulties and more than 70% reported non-white ethnicity.

## 2. Method

### 2.1. Participants and Procedures

The present study was part of the project entitled *Blurred Lives*, conducted over two years (2017–2019) by five European partners and funded by Erasmus+ (for a detailed description of the project, see [17,58]). The project envisaged the collaboration between researchers from five countries and included adolescents belonging to schools selected in areas of socio-economic disadvantage. The criteria for selecting schools were different for each country (for an in-depth description, see [58]). In Italy, for example, schools were selected based on their educational pathways—i.e., technical or vocational—and on socio-economic data officially provided for each Italian school by the Ministry of Education (i.e., “Scuola in Chiaro”; transl. “School made Clear”); in England, other criteria such us the percentage of students entitled to free school meals were considered.

The main objective of the Blurred Lives project was to create, using a co-participatory approach, resources for parents, teachers, social network providers, and young people, aimed at raising awareness of the issue of cyberbullying. In the present article, we analyzed some of the resources produced by young people for their peers.

The participants were 59 early adolescents (52.5% male) across five countries, aged between 14 and 16 years. The participants were divided into 11 groups, each of which consisting of a number of adolescents ranging from 3 to 9 members (*M* = 5.36, *SD* = 1.96): two groups from two schools in England; two groups from two schools in Germany; three groups from three schools in Italy; two groups from one school in the Netherlands; and two groups from two schools in Northern Ireland. During school hours, students in each group were asked to participate in Quality Circles (QCs; [58,59,60]), that is, co-participatory group sessions based on three phases. In phase one, participants were encouraged to analyze the issue of cyberbullying and to identify some examples, along with their possible causes and solutions. In phase two, participants translated their ideas into comics to share with a wider audience of young people from all over Europe. In phase three, participants presented their outputs to schoolmates and, when possible, to a broader audience for consideration. Overall, QCs lasted approximately two months and were divided into seven weekly sessions, each lasting about one hour, with differences in implementation in the five countries due to the need to adapt the program to each school (for a more in-depth discussion of the procedures used in the Quality Circles and of the specific measures adopted in the five Countries, see [17]). No attrition of program participants occurred, although occasionally a few students were unable to attend a QC session due to sporadic absences from school.

During all sessions, a researcher-facilitator was present to explain to students the objectives of QCs, encourage participation, and facilitate task orientation when necessary. Nevertheless, researchers’ intervention was reduced to the minimum, and participants were left free to decide the contents of their work, the division of tasks, and the procedures to be adopted [17]. By and large, most participants worked productively and collaboratively. However, facilitators from different countries reported a few difficulties, mainly related to the lack of attention and participation of some group members, and to disagreement in relation to the contents to be included in the comics (for a detailed description of these issues, see [17]). Overall, 10 comics were produced by nine groups of young participants (one British group created two comics). Each comic can be found at the link https://www.ou.nl/web/blurred-lives/resources-teens (accessed on 25 May 2022). In Table 1, we provide a list of the produced comics reporting their IDs, titles, and contents. No comic was produced by the participants from Northern Ireland, as they preferred to create different types of resources (i.e., informative material on cyberbullying in the form of illustrated posters).

Participants were informed about the project goals, the confidentiality of their work, and the voluntariness of participation. Informed consent from parents was also collected before the beginning of QC sessions, with no family refusing. The study was conducted in agreement with the ethical guidelines for protecting human participants. Each of the five European partners involved in the project gained the approval of their local Ethics Committee.

### 2.2. Coding Procedures

The collected comics were analyzed for their content by considering text (e.g., speech bubbles and captions) and visual (e.g., setting representation, characters’ emotional facial expressions) information. The coding system was defined by building on an inductive-deductive methodology [61,62]. In the first phase, an inductive method was used by two researchers (first and last author of the article) who, first individually and then collaboratively, considered the comics and identified the main emergent themes within them. In the second phase, a deductive method was used to link the identified themes to the results and categorizations provided by the literature in previous studies.

Codes were organized hierarchically and included four primary themes: cyberbullying episodes, coping strategies, characters, and emotions. Cyberbullying episodes were coded accordingly to the type of aggression, the platform where the event occurred, and their possible connection with forms of traditional bullying [63]. It was possible to code multiple cyberbullying incidents in the same comic. The coding system is detailed in Table 2.

Regarding coping strategies (Table 2), we coded the presence of cognitive, confrontational, passive, and social strategies, as suggested by several authors [52,64,65]. We also added suicide as an extreme consequence of cyberbullying. It was possible to code different coping strategies in the same comic.

For each character present in the comics, we coded their role (cyberbully, cybervictim, reinforcer, bystander, defender [53]), gender (boy, girl, unknown), and peer or adult group membership (peer, parent, teacher, another adult, unknown). Then, to evaluate the relevance of each character, we counted how many times they appeared in the vignettes (e.g., the same character in the role of the cyberbully could appear two or more times in the cartoons).

Finally, we focused on identified primary emotions (joy, surprise, disgust, sadness, anger, fear [42,66,67]), as they are universally experienced in all human cultures and are easily recognizable by facial expression indicators. Unfortunately, the analysis of amateur comics did not allow us to identify reliable indicators of more complex emotions, such as shame, or assess the intensity of identified emotions. We annotated the characters’ roles and how they were represented (text, drawing, text and drawing) by using NVivo software (QSR International Pty Ltd., 2008). NVivo can organize a great deal of information related to a source (such as a comic) and has a strong coding function, allowing researchers to quickly capture the information points contained in the material [68]. In our case, we formed six main categories, representing each primary emotion. For each of them, we created: a) five subcategories representing different roles (cyberbully, cybervictim, defender, reinforcer, bystander), and b) three subcategories describing how the emotion was expressed (text, drawing, text and drawing).

Each comic was analyzed by two independent coders (the first and last author of the manuscript). Intercoder reliability (Cohen’s kappa) was calculated for each category analyzed and ranged from 0.856 to 1.00.

### 2.3. Data Analysis

Given the primarily exploratory nature of this study, we calculated descriptive statistics for the coded variables. Concerning cyberbullying incidents (type, platform, association with traditional bullying), no statistical analyses were carried out due to the low number of units of analysis. One-sample Chi-Square analyses were run to verify the distribution of coping strategies, as well as of gender and group membership for each role. Furthermore, we used Kruskal-Wallis H tests to verify differences among characters’ roles with respect to their relevance in vignettes and experienced emotions. Finally, a one-sample Chi-Square analysis was used to test the distribution of emotions in the three categories of drawing, text, and drawing and text.

## 3. Results

### 3.1. Cyberbullying Incidents

Twelve cyberbullying incidents (see Table 3) were represented in the comics, with two of them (ID 7 and 8) involving two episodes each. The types of cyberbullying depicted were denigration (58.3%), followed by direct unpleasant comments (25%), sexting (8.3%), and the creation of a fake account (8.3%). Cyberbullying incidents occurred on social media (58.3%), private chats (16.7%), and gaming websites (16.7%). In seven (58.3%) out of twelve incidents, cyberbullying episodes were associated with traditional forms of bullying.

### 3.2. Coping Strategies

Overall, we identified 26 coping strategies (see Table 3). Social coping (38.5%) was depicted in each comic several times, involving different actors. We also identified passive (23.1%) and confrontational (23.1%) coping, represented in six comics, and cognitive coping (7.7%), represented in two comics. Suicide (7.7%) was portrayed in two comics in terms of suicidal ideation and committed suicide. No difference in the distribution of coping strategies was found, χ^2^(4, *N* = 26) = 8.62, *p* = 0.071.

### 3.3. Characters

#### 3.3.1. Presence of Roles, Gender, and Peer or Adult Group Membership

Overall, we identified the presence of 154 characters (see Table 4). All the comics were characterized by at least one cyberbully (*n* = 13) and cybervictim (*n* = 11). Reinforcers (*n* = 55) and defenders (*n* = 22) were represented in seven and nine comics, respectively. In three out of ten comics, we identified bystanders (*n* = 53); however, 50 of them were represented in one comic, indicating a large audience. In general, the distribution of characters showed higher frequency in the role of reinforcers and bystanders, χ^2^ (4, *N* = 154) = 60.54, *p* < 0.001. As reported in Table 4, cyberbullies’ gender was not codable in 28.5% of cases, while they were depicted as males in 46.1% of cases and females in 15.4% of cases. We did not perform a chi-square test because for three cells (100.0%) the expected values were lower than 5. Cyberbullies were depicted as peers (61.5%) or their group membership was unknown (38.5%), χ^2^(1, *N* = 13) = 0.69, *p* = 0.41. The gender distribution of cybervictims did not differ significantly between girls (63.6%) and boys (36.4%), χ^2^(1, *N* = 11) = 0.82, *p* = 0.37. Cybervictims were always represented as peers. Reinforcers’ gender was mainly unknown (83.6%), followed by a small number of characters represented as males (3.6%) or females (12.7%), χ^2^(2, *N* = 55) = 63.31, *p* < 0.001. Furthermore, they were depicted as peers (60%) or without specifying group membership (40%), with no difference in distribution regarding group membership, χ^2^(1, *N* = 55) = 2.20, *p* = 0.14. Defenders were equally distributed between males and females (45.5%), with the gender not specified in only 9.1% of cases, χ^2^(2, *N* = 22) = 0.5.82, *p* = 0.05. They were mainly represented as adults (parents 45.5%; teachers 13.6%; other adults—e.g., school principals or police officers—18.2%), although peers (22.7%) were also represented, χ^2^(1, *N* = 22) = 6.55, *p* = 0.01. Concerning bystanders, gender was unknown in most cases (98.1%), and was specified as male in only one case (1.9%), χ^2^(1, *N* = 53) = 49.07, *p* < 0.001. They were mainly depicted without specifying group membership (94.3%), and as peers (5.7%) in only a few cases, χ^2^(1, *N* = 53) = 41.68, *p* < 0.001.

#### 3.3.2. Relevance of Characters

Comics were made up of several framed illustrations, ranging from 8 to 21, and totaling 123 vignettes. Overall, cybervictims were the most represented, as they were included in 78 vignettes (63.4%). Defenders were depicted in 41 vignettes (33.3%), cyberbullies in 30 (24.4%), reinforcers in 17 (13.8%), and bystanders in 4 (3.3%). The Kruskal-Wallis H test showed a statistically significant difference in the relevance of characters’ roles, *H*(4, *N* = 154) = 102.09, *p* < 0.001, with a mean rank score of 146.91 for cybervictims, followed by 109.05 for defenders, 107.04 for cyberbullies, 61.90 for reinforcers, and 60.72 for bystanders. Non-parametric in-pairs comparison post-hoc tests were significant (*p* < 0.001), except for reinforcers-bystanders (*p* = 0.85) and cyberbullies-defenders (*p* = 0.86).

### 3.4. Emotions

#### 3.4.1. Identified Emotions and Emotions by Role

A total of 87 emotional expressions were identified in our comics, in the following frequency order: sadness (*n* = 36; 41.4%), joy (*n* = 32; 36.8%), surprise (*n* = 10; 11.5%), anger (*n* = 7; 8%), and fear (*n* = 2; 2.2%). We did not identify disgust in any of the comics.

**Figure 1:**
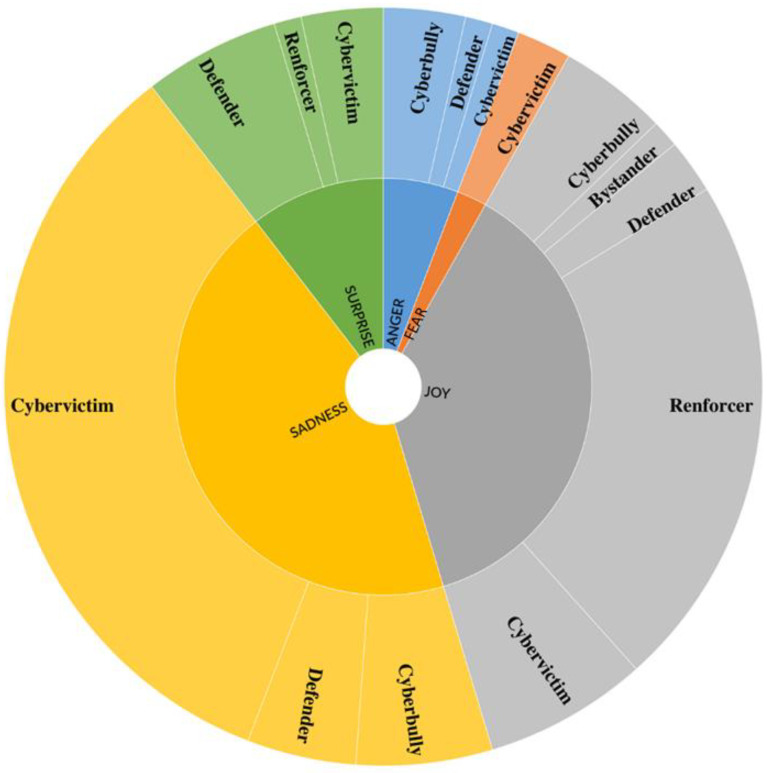
Emotions by role. The innermost circle represents the emotions; the external circle the roles for each emotion.

Overall, as shown in Figure 1, emotional expressions were associated with cybervictims *(n* = 43; 49.4%), followed by reinforcers (*n* = 21; 24.1%), cyberbullies (*n* = 11; 12.6%), defenders (*n* = 11; 12.6%), and bystanders (*n* = 1; 1.1%). The Kruskal-Wallis H test showed a statistically significant difference in the emotional expressions of characters’ roles, *H*(4, *N* = 154) = 58.94, *p* < 0.001, with a mean rank score of 144.45 for cybervictims, followed by 82.73 for defenders, 81.54 for reinforcers, 81.31 for cyberbullies, and 56.31 for bystanders. Non-parametric in-pairs comparison post-hoc tests revealed that emotions were significantly more represented in cybervictims compared to all the other roles (*p* < 0.001 for all the comparisons). In addition, emotions in reinforcers and defenders were significantly more frequent compared to bystanders (*p* = 0.002 and *p* = 0.035, respectively).

Given the low number of single emotions, no statistical analysis was computed for these variables in relation to their distribution among the five roles (cyberbullies, cybervictims, reinforcers, defenders, and bystanders). Descriptively analyzing emotions related to the different roles, sadness was associated with cybervictims (*n* = 29; 80.6%), followed by cyberbullies (*n* = 5; 13.9%) and defenders (*n* = 2; 5.6%), in different scenarios. Indeed, cyberbullies’ sadness was almost exclusively depicted in the last vignettes of the comics, when they were reprimanded by parents or teachers for their actions and realized the damage caused to cybervictims. By contrast, defenders manifested sadness when witnessing the cyberbullying incident or when victims were asking for help. Students represented the emotion of joy in association with each role: reinforcers (*n* = 20; 62.5%), cybervictims (*n* = 6; 18.7%), cyberbullies (*n* = 3; 9.4%); defenders (*n* = 2; 6.3%), and bystanders (*n* = 1; 3.1%). While reinforcers were represented laughing at the victim, cybervictims expressed joy at the beginning of the comics, when the cyberbullying episode had not yet occurred, or at the end, as an emotional response to the incident resolution. Anger was represented in relation to cybervictims (*n* = 2; 28.6%), defenders (*n* = 2; 28.6%), and cyberbullies (*n* = 3; 42.9%). While cybervictims’ and defenders’ anger was directed towards the cyberbully, cyberbullies’ anger represented a response to external variables (e.g., a storm that cut off electricity to the house, making it impossible to play online). Fear was only associated with victims (*n* = 2; 100%) as a reaction to cyberbullying incidents. Finally, surprise was portrayed in relation to cybervictims (*n* = 4; 40%), defenders (*n* = 5; 50%), and reinforcers (*n* = 1; 10%). It is worthwhile noting that defenders’ surprise was in all cases manifested by adults (such as parents and teachers) when discovering what had happened.

#### 3.4.2. Representation of Emotions

**Figure 2:**
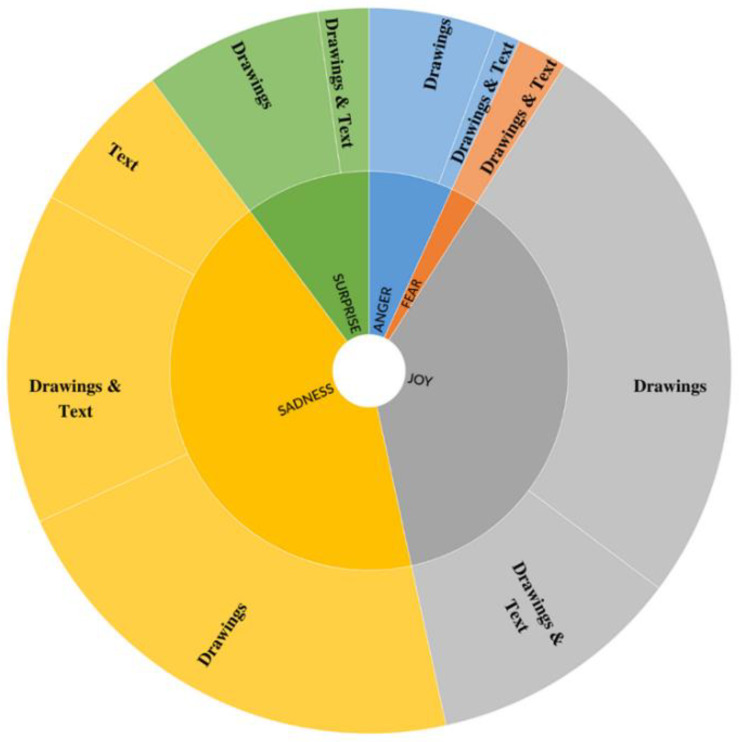
Emotions by visual and text representations. The innermost circle represents the emotions; the external circle is the visual representation for each emotion.

**Figure 3:**
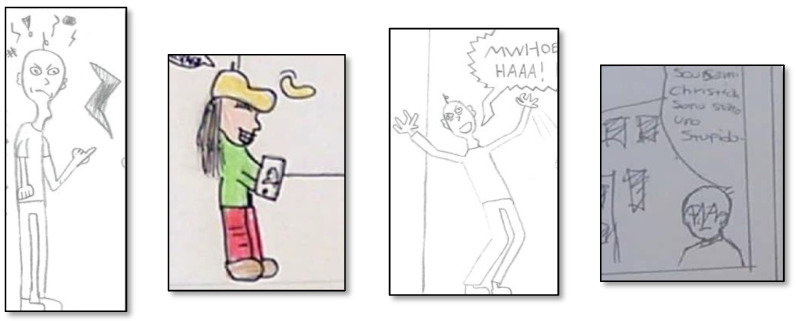
Examples of cyberbullies’ emotions. From left to right: Anger, Joy, Joy, Sadness. The phrase “Scusami Christian sono stato uno stupido” means “Sorry Christian, I was a stupid”.

**Figure 4:**
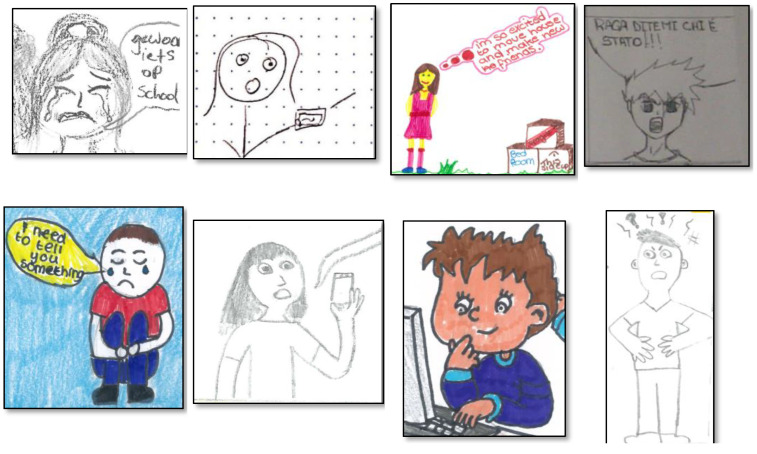
Examples of cybervictims’ emotions. From left to right in both rows, Sadness, Surprise, Joy, Anger. The phrase “Gewwon iets op school” means “Just something at school”; the sentence “Raga ditemi chi è stato!!!” means “Guys tell me who did it!!!”.

**Figure 5:**
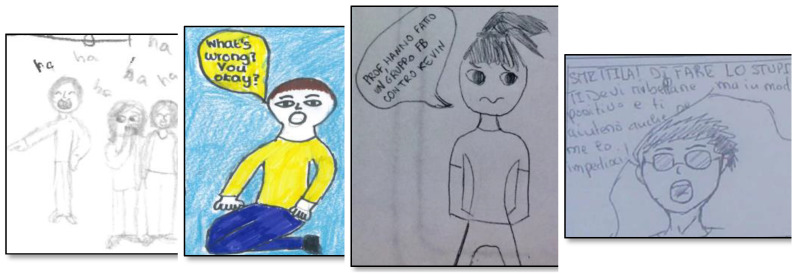
Examples of other roles’ emotions. From left to right: Joy (reinforcer), Surprise (defender), Sadness (defender), Anger (defender). The phrase “Prof. hanno fatto un Gruppo FB contro Kevin” means “Prof., they created a FB group against Kevin”; while the sentence “Smettila di fare lo stupido. Ti devi ribellare ma in modo positivo e ti aiuterò anche se me lo impedisci!” means “Stop being stupid. You have to rebel but in a positive way and I will help you even if you stop me!”.

Emotions were mainly represented using visual information (*n* = 48; 55.2%) or combining text and images (*n* = 33; 37.9%), while the use of only text (*n* = 6; 6.9%) was less frequent, χ^2^ (2, *N* = 87) = 31.35, *p* < 0.001. Given the low number of single emotions, no statistical analysis was computed for these variables in relation to their distribution in the three categories drawing, text, and drawing and text. As shown in Figure 2, sadness was represented by drawings (*n* = 18, 50.0%), drawings with text (*n* = 12, 33.3%) and text (*n* = 6, 16.7%). Cybervictims, for instance, were often depicted crying, and text was usually used to reinforce the emotion with sentences like “I do not know what to do”, or “I can’t tell anyone”. Joy was represented by drawings (*n* = 19, 59.4%) and drawings with text (*n* = 13, 40,6%). When joy belonged to the reinforcers, it was often accompanied by insults toward the cybervictim like “jerk”, “loser”, or “hahaha” laughter. Concerning anger, it was represented by drawings (*n* = 5, 71.4%) and drawings with text (*n* = 2, 28.6%). When anger was expressed only through drawings, the characters were represented with, for instance, angry faces or thunderbolts drawn above their heads. Surprise, represented with drawings (*n* = 7, 70%) and drawings with text (*n* = 3, 30%), was mainly depicted by characters with open mouths, and accompanied by expressions like “oooh”. Finally, fear was represented only by drawings and text (*n* = 2, 100%), with sentences like “I’m afraid, I can’t do it”. Examples of emotions for cyberbullies, cybervictims, and other roles, using visual and textual elements, are shown in Figure 3, Figure 4 and Figure 5.

## 4. Discussion

The main aim of this study was to explore how early adolescents from disadvantaged socio-economic backgrounds represent the phenomenon of cyberbullying to their peers through comics using a co-participatory approach. To do so, we analyzed 10 comics—produced by adolescent participants—in terms of cyberbullying incidents, coping strategies, characters, and emotions. Key findings and practical implications are discussed in detail in the following sections.

### 4.1. Cyberbullying Incidents

In line with our expectations and consistent with the literature, our participants illustrated cyberbullying as a multifaceted phenomenon, both in terms of types [1,46] and platforms [47]. In particular, adolescents decided to depict, in over half of the episodes, examples of denigration, followed by unpleasant comments. These results suggest that adolescents are aware of which types of cyberaggression are more frequent and confirm verbal violence as the most common type of cyberbullying [1]. At the same time, adolescents illustrated other forms of cyberbullying, such as sexting and creating fake accounts, that, even if less frequent, are particularly severe in terms of their legal implications and adverse effects on victims, suggesting the need for prevention and intervention [69].

Concerning online platforms, students situated cyberbullying incidents on social media in seven out of twelve episodes, thus confirming that the type of platform used to perpetrate acts of aggression is the one most used by adolescents in their everyday experience [70]. In a few comics, adolescents also depicted aggressive behavior on other platforms such as private chats or gaming websites, suggesting that cyberbullying can happen in any online venue where virtual interaction is possible [1].

Finally, in over half of the episodes, cyberbullying was associated with traditional forms of bullying, confirming a solid continuity among these phenomena [50,70]. In general, aggressive behavior started in the online environment, such as in the *Snap Attack* comic, but denigration continued in class with classmates who verbally bullied the victim, amplifying the reiteration and the impact of the aggression. In another comic, *We’re like books… Who goes beyond the cover?* again the aggression started online, and it was followed by physical bullying, suggesting that cyberbullying shows a strong continuity not only with verbal but also with physical bullying. Although these results were expected and are in line with the literature, they represent noteworthy findings considering that they emerged from a new methodological approach.

### 4.2. Coping Strategies

As suggested by Perren et al. [71], to better understand coping strategies, in-depth qualitative studies are recommended and helpful. In line with previous research [51,52,71] and our expectations, we found several coping strategies depicted in the comics. Nevertheless, more interesting for our purposes is the complex interplay between them. In nine out of ten comics, students illustrated an escalation of coping strategies, generally from passive or confrontational strategies to social ones. In this respect, we can propose two possible explanations. First, our participants emphasized with their comics that cyberbullying is unlikely to be effectively addressed at the first attempt, thus normalizing the need for a trial-and-error approach in which a range of coping strategies of varying effectiveness can be considered. In the comic *Cyberbullying according to us*, for instance, the first confrontational coping strategy leads to the victim’s desperation; it is only by asking for help that the victim was able to find an effective solution. Second, the main message presented by young people to their peers is that cyberbullying can be stopped without losing hope and with the support of other people. In the comic *Ask for help if you’re being bullied*, for example, participants gave a double ending characterized by two coping strategies: the first ending involved the victim’s suicide, while in the second ending the victim, through asking for help, managed to recover. Indeed, our analysis of the comics shows that social coping, involving peers and especially adults, was the most effective coping strategy to tackle cyberbullying. It is noteworthy that in several comics, peers or adults, realizing that something was wrong, offered their help. It is also remarkable that social coping often involved the combined intervention of different actors: in the comic *Everyone can do something*, for instance, the victim asks a peer for help; then, the peer suggests asking for help from parents, who in turn ask for support from the police and a psychologist. This complex representation of social coping—which would have been difficult to achieve through other methodological approaches—seems to indicate, from adolescents’ perspective, that not only is cyberbullying a social phenomenon [12] but so too is its resolution process. The adolescents were aware that only when the community works together is it possible to address cyberbullying episodes effectively, and as suggested by a recent review [1], joint efforts are required from students, parents, and schools, to create a cyberbullying-free environment.

The last consideration concerns suicide: in the comics, adolescents seem to suggest that passive coping strategies, up to severe self-harming, are the only solution when social support is not present, stressing how important it is to ask for and receive help. This result confirms that suicide is considered by teens as an extreme answer for cybervictims, as suggested by reviews on the effect of victimization on psychological wellbeing [4,5]. This message is particularly alarming as it comes from students from areas of socio-economic disadvantage. As suggested by the literature, adolescents from disadvantaged socioeconomic backgrounds are less satisfied with their lives, report lower levels of mental wellbeing [72], and are more at risk of suicide [73].

### 4.3. Characters

Concerning characters, we found some differences in terms of presence and relevance. The analyses focused on presence revealed that the roles represented in our comics overlap with the roles involved in cyberbullying already described in the literature [53], with higher numbers of reinforcers and bystanders, thus confirming the perception of cyberbullying as a social event [12]. Concerning the relevance, although the roles of cyberbully and cybervictim were the only ones present in all our comics, cybervictims and defenders appeared more often in the vignettes. These results are only partially consistent with previous studies that considered cyberbullies and cybervictims the most important cyberbullying actors [51]. Probably to sensitize a wide young audience to cyberbullying, our participants decided to emphasize not only the role of the cybervictim but also the role of the defender, presumably perceived as crucial to disrupting the flow of aggression. The relevance of defenders is consistent with the enhancement of social coping discussed above and strengthens the message that cybervictims need help to save themselves.

Concerning bystanders, although this role was played by several characters, as revealed by the analysis of presence, it did not seem crucial in the storytelling, as shown by the analysis of relevance. Indeed, bystanders were depicted in only 4 out of 123 vignettes. Nonetheless, in one vignette more than 50 bystanders with stylized faces appeared, suggesting that, when present, this role assumes the form of a large and indefinite audience that, as already described in the literature [74], can impact the severity of the phenomenon.

Concerning gender, students represented both males and females in each role, thus communicating to their peers that all are involved in cyberbullying, even if with slight differences, in line with the literature [1,54,55]. Regarding group membership, students represented cyberbullies, cybervictims, and reinforcers as peers, or as characters with an unknown identity (especially for reinforcers), and never as adults. By contrast, adults were overrepresented in the role of defender. With these choices, students seem to stress that even if cyberbullying is a phenomenon among peers, they need the intervention of adults to solve the situation, as already highlighted in relation to coping strategies. Lastly, it is particularly relevant that adolescents did not specify gender and group membership of several characters, thus confirming anonymity as one of the key aspects distinguishing cyberbullying from bullying [70,75], and as a factor that can impact its severity [74].

### 4.4. Emotions

Taking a step forward with respect to previous studies—mostly focused on the emotional impact of cyberbullying on bullies and especially victims [56], and on their moral emotion patterns [57]—our study has shed new light on the multiplicity of emotional experiences involved in cyberbullying. The comics in our study, with more than 80 registered emotions, have confirmed an image of cyberbullying as a highly emotional phenomenon, including a variety of positive and especially negative emotions attributed to different roles involved. Although nearly 37% of the recorded emotions expressed joy, most of them were negative, and unsurprisingly, sadness was the most recurrent coded emotion. Even for our small sample, this finding confirms a vision of cyberbullying as a highly harmful phenomenon, bursting into young people’s lives with negative affective effects [76].

Sadness was associated with cybervictims in more than 80% of cases, and fear in all of the cases, in line with the literature [77,78]. Nonetheless, sadness was also associated with defenders, possibly indicating empathy towards the victim, and cyberbullies. Given that cyberbullies’ sadness came commonly after discovering their actions, this emotion could be interpreted as an outcome of the social disapproval of their aggressive behaviors [79]. Anger was depicted in relation to cybervictims and defenders, plausibly indicating a negative activation in response to the unpleasant event and a willingness to restore a desired condition [77,80]. Cyberbullies’ anger, on the other hand, was represented as a reaction to uncontrollable events (e.g., a thunderstorm), as if adolescents perceive that this role is somehow characterized by emotional dysregulation, as confirmed by previous studies [81]. Surprise was associated with cybervictims and defenders, possibly suggesting a first reaction of dismay and shock to what happened, and was related to reinforcers in only one case. Finally, joy was associated with all the roles involved in cyberbullying. As far as cyberbullies and reinforcers are concerned, this emotion—which could most likely invoke feelings of pride—could be interpreted as a sign of moral disengagement in relation to harmful behaviors, in line with the literature [57,71]. Concerning cybervictims and defenders, it is noteworthy that positive emotions were depicted mainly at the beginning or the end of the comics, possibly to stress the transition from a condition of wellbeing (before the cyberbullying episode) to one of distress (after the incident), again to one of wellbeing (once the incident had been addressed effectively and the perpetrator identified and punished). By and large, these results are particularly innovative, adding new considerations about the relations between cyberbullying roles and emotions.

Finally, characters’ emotional states were mainly represented through drawings or the combination of drawing and text, and only rarely through text alone. This result can be mainly interpreted in the light of our methodological choices, i.e., using comics-based research, which by nature implies multimodal information with visual elements generally prevailing over textual ones. Notwithstanding this, as highlighted by previous studies on populations at high risk and with linguistic difficulties [29,38], the possibility of expressing thoughts and emotions with additional channels to the verbal one represents an important opportunity to grasp young people’s nuanced perceptions and understanding of cyberbullying.

## 5. Limits

The study presented in this paper has some limits that should be considered when interpreting the results and for future research. First, our study was exploratory, and the generalizability of our findings is undoubtedly limited due to the unique methodology we used and the small number of participants. Although the collected comics presented a complex and innovative picture of adolescents’ understanding of cyberbullying—and especially of what they consider important to emphasize to their peers—a larger sample would have provided more reliable information, and a larger amount of data would have allowed more sophisticated statistical analyses. Furthermore, the possibility of combining a comics-based method with other instruments should be carefully considered in future works. This seems particularly relevant for the interpretation of emotions: in this study, we attempted to identify primary emotions remaining as close as possible to textual and visual indicators, and within this process, we inevitably reduced the complexity of the emotional experiences that adolescents associate with cyberbullying. For example, it is highly plausible that the sadness we coded in association with cyberbullies could take the form of guilt or shame, while the sadness associated with cybervictims and defenders could be related to more complex experiences of anxiety, shame, or despair [57,82]. Interviews and focus groups aimed at deepening the understanding of the artifacts produced would have been useful to distinguish between different and more multifaceted emotional expressions.

Second, in this study, we chose to work with adolescents from disadvantaged backgrounds, and we cannot know whether, and to what extent, the collected comics were affected by this dimension, nor if we would have achieved different results with teenagers from more affluent backgrounds. In this respect, previous results from the literature are inconsistent. On one hand, the latest Health Behaviour in School-aged Children (HBSC) survey, involving over 50 countries and regions across Europe and Canada, found no clear associations between family-income levels and cyberbullying, suggesting that violence in adolescence is not connected with socioeconomic background. Conversely, other studies conducted on bullying phenomena [83] suggested that “feelings of shame, humiliation and distrust intensify with greater income inequality and create a harsh social environment where violent acts such as school bullying may be condoned or ignored” (p. 357). Given these mixed results, future studies using arts-based methods should contemplate the possibility of comparing artifacts produced by adolescents from different socioeconomic backgrounds.

Third, in this study participants worked with researchers to produce comics to be freely disseminated to a large audience of teenagers from all over Europe and beyond. Indeed, to make the comics as engaging and understandable as possible, each of them has been translated into four languages (English, German, Dutch and Italian) and redesigned by a professional cartoonist. Nonetheless, we do not have any information on how effective these cartoons are, or have been, in engaging and helping a wider audience of young people to learn more and tackle the issue of cyberbullying.

## 6. Conclusions

Notwithstanding the limitations discussed above, this study provides insights into scientific, practice, and policy frameworks. First, by taking a step forward compared to the previous literature, our study emphasizes the importance of adopting innovative co-participatory methods to gain a richer, deeper understanding of cyberbullying. The decision to rely on comics-based research [30] allowed us to collect multimodal products—where images enrich the text, and vice versa—and to mirror the subjectivity of those who produced them. On the one hand, our findings confirm—using a new methodology—some considerations already described in the literature, such as that cyberbullying is a social phenomenon, including different roles, types of behaviors, and platforms, and involves a strong continuity with traditional bullying. On the other hand, comics suggest two elements of novelty that would be difficult to capture using other adult-centered methodological approaches. The first one is the articulated trial-and-error coping pathways that young people depicted to deal with cyberbullying, with asking for support from adults or peers as the final and most effective solution to the phenomenon. The relevance of defenders confirms the importance of social support in all comics in dealing the cyberbullying. The second element of originality is the representation of cyberbullying as an emotion-filled phenomenon. The comics shed light on the positive and negative emotional experiences involved in cyberbullying, with specificity in each role.

To conclude, it could be useful to dwell briefly on a few procedural considerations highlighting the weaknesses, but also the strengths, of the co-participatory approach, used. On the one hand, working in groups to produce comics was not always simple for our participants (see [17]), as some students struggled to pay attention and actively participate. As QCs were not combined with individual interviews, we cannot know whether this was because some adolescents found the task boring, or because they felt uncomfortable engaging with their peers on sensitive (and maybe personally confronting) topics such as cyberbullying. In future studies adopting co-participatory methods, scholars should carefully reflect on these issues, and consider providing additional confidential settings to make participants more comfortable in revealing their ideas and experiences. On the other hand, as detailed by Hamilton and colleagues [17], most of the participants enjoyed QCs and showed satisfaction in realizing the outputs. In the shift from being research objects to playing an active co-researcher role, students not only had the chance to personally reflect on topics that are of close interest to them in their daily lives, but they also felt part of the solution and empowered to proactively contribute their own knowledge and opinions in providing practical help to their peers. This study thus emphasizes that it is possible to give a voice to students from areas of socio-economic disadvantage, using additional channels beyond the verbal one, and actively involve them to reflect on (and promote change in) issues from which they should not be left out. Moving forward, we must listen to their voices and translate their understanding and insights into effective practice, taking into account the multiple emotions involved, and aimed at increasing social support, especially from adults, in dealing with cyberbullying.

## Figures and Tables

**Table 1:** Comic IDs, Country origin, titles, and contents.

Comic IDs and Country	Titles and Contents
ID 1, England	*All or nothing* Hunter is severely insulted online for how he plays Fortnite. At first, he is very sad, he does not want to talk to anyone, but the insults continue. At that point, Hunter decides to ask for help from his father who gives him very useful advice on how to defend himself online.
ID 2, England	*Blue hearts* Hope moves and changes schools, and she is very excited about it. After the first classes, at home, Hope starts playing Fortnite but, as she plays like a beginner, she receives several insults online. Even at school, things go differently from how she imagined, and in the classroom, she is teased. Sad and depressed, Hope thinks about suicide, but then starts to think of the positive things in her life. She realizes that she is not alone and that there are several resources, like this comic, which can help her.
ID 3, England	*Snap attack* Hope uploads a photo of herself on her Snapchat profile, and two peers write to her privately asking for pictures of her naked. Hope sends them naked pictures, which are suddenly shared online by the two boys. Hope’s classmates insult her and make fun of her online and offline. Hope reports the incident to her mother, who immediately decides to talk to the teachers. In the end, a school meeting about bullying and cyberbullying takes place.
ID 4, Germany	*Stop cyberbullying* A teacher suggests to his students that they should create a comic about cyberbullying. The pupils do not actually like this idea, and they decide to create an Instastory instead, with smart tips to address cyberbullying. One of the girls in the class, a victim of bullying, observes online the contents created by her classmates and uses these tips to deal with her problems.
ID 5, Germany	*Everyone can do something* After posting a picture on Instagram, Ella receives hateful messages, and even at school people laugh and insult her. Ella deletes the picture from Instagram and reports the bullies. However, the distribution of the picture cannot be stopped: it has already been shared by screenshots via WhatsApp. As suggested by her friend Danielle, Ella asks her parents for help, and they decide to report what has happened to the police. Ella asks a therapist for help.
ID 6, Italy	*Cyberbullying according to us* An anonymous user uploads onto social media an embarrassing image of Christian, who is teased both online and at school. Aldo, one of Christian’s friends, offers him some help. Initially Christian says he does not need it, but then, encouraged by his friend, he decides to talk about the cyberbullying episode with his parents, who in turn talk to the school principal and contact the Police. The Police identify the cyberbully, Giovanni, who admits his mistake and apologizes.
ID 7, Italy	*No to bullies* In the school toilets, some female students shove a girl, record her while she is crying, and upload the video onto social media. Other girls watch, indifferent or amused, and only one girl tries to stop them. On social media, the video is commented on by other young people, who insult and make fun of the victim. A girl tries to convince the victim to talk to the teacher, who discusses the incident with the whole class. The bullies understand what they did and apologize.
ID 8, Italy	*We’re like books…who goes beyond the cover?* Kevin chats with an anonymous user to whom he reveals personal information. The next day, at school, he is teased by everyone, and he discovers from his friend Claire that someone has created a Facebook page about him. Claire convinces Kevin to talk to the teacher, who has a meeting with his parents at school. Kevin and Claire discover that the cyberbully is Michael; they go to talk to him, but the discussion degenerates into a brawl. In the final scene, Michael receives the expulsion letter from the school.
ID 9, The Netherlands	*Ask for help if you’re being bullied* Sarah is teased and insulted on social media and at school. The story has two alternative endings. In the first, Sarah does not talk to anyone and decides to commit suicide. In the second, Sarah reveals to her mother that she is a victim of cyberbullying; they go together to the police, who solve the problem by directly contacting the bullies.
ID 10, The Netherlands	*The anonymous moles* Harry gets bored because he cannot play video games as usual, and he decides to spend time insulting other young people online. The first victim (Noah) reacts angrily, and this triggers a heated discussion between the two. Mara, the second victim, ignores Harry’s insults and reports what happened to her parents, who contact Harry’s mother. The cyberbully’s mother argues with him, explaining that his behavior is not acceptable.

**Table 2:** Coding system of cyberbullying episodes and coping strategies.

Categories	Subcategories and Description	References
Types	*Denigration*: Embarrassing photos, videos or personal information relating to the victim were edited, posted, shared, tagged or commented without victim’s permission. *Direct unpleasant comments*: one or more offensive or nasty messages were directly sent to the victim *Sexting*: Asking, sending, receiving, and forwarding sexual images. *Fake accounts*: a fake account of the victim was set up by someone.	Adapted from [63]
Platforms	*Social media*: cyberbullying took place on social platforms such as Instagram or Snapchat. *Private chat*: cyberbullying took place in a private chat (e.g., WhatsApp) between two or more people. *Gaming website*: cyberbullying took place on a gaming website (e.g., Fortnite). *Other—Not specified.*	Adapted from [63]
Coping strategies	*Cognitive*: the victim uses mental strategies to deal with the emotional harm caused by one or more cyberbullying episodes (e.g., thinking optimistically). *Confrontational*: the victim takes an action aimed at directly stopping cyberbullying (e.g., s/he blocks the cyberbully on social media, creates a new account, changes the password or searches for a confrontation). *Social*: the victim seeks help from a peer or an adult. *Passive*: the victim does nothing and just hopes that cyberbullying will stop. *Suicide*: the victim has suicidal thoughts or commits suicide.	[51,52,64,65]

**Table 3:** Types of cyberbullying, platforms, association with traditional bullying, coping strategies.

Cyberbullying Episodes	*n*	*%*
Type		
Denigration	7	58.3
Direct unpleasant comments	3	25.0
Sexting	1	8.3
Fake accounts	1	8.3
Total	12	100.0
Platform		
Social media	7	58.3
Private chat	2	16.7
Gaming website	2	16.7
Other—Not specified	1	8.3
Total	12	100.0
Association with traditional bullying		
Yes	7	58.3
No	5	41.7
Total	12	100.0
Coping strategies		
Cognitive	2	7.7
Confrontational	6	23.1
Social	10	38.5
Passive	6	23.1
Suicide	2	7.7
Total	26	100.0

**Table 4:** Characters in terms of role, gender, and peer or adult group membership.

Variables	Cyberbully		Cybervictim		Reinforcer		Defender		Bystander	
	*n*	*%*	*n*	*%*	*n*	*%*	*n*	*%*	*n*	*%*
Gender										
Male	6	46.1	4	36.4	2	3.6	10	45.5	1	1.9
Female	2	15.4	7	63.6	7	12.7	10	45.5	0	0.0
Unknown	5	38.5	0	0.0	46	83.6	2	9.1	52	98.1
Total	13	100	11	100	55	100	22	100	53	100
Group membership										
Peer	8	61.5	11	100.0	33	60.0	5	22.7	3	5.7
Parents	0	0.0	0	0.0	0	0.0	10	45.5	0	0.0
Teacher	0	0.0	0	0.0	0	0.0	3	13.6	0	0.0
Other adults	0	0.0	0	0.0	0	0.0	4	18.2	0	0.0
Unknown	5	38.5	0	0.0	22	40.0	0	0.0	50	94.3
Total	13	100	11	100	55	100	22	100	53	100

## Data Availability

The data presented in this study (e.g., codings) are available on request from the corresponding author. All the original comics can be retrieved from the following link: https://www.ou.nl/web/blurred-lives/resources-teens (accessed on 25 May 2022).
